# Review of databases for experimentally validated human microRNA–mRNA interactions

**DOI:** 10.1093/database/baad014

**Published:** 2023-04-25

**Authors:** Dorian Kariuki, Kesava Asam, Bradley E Aouizerat, Kimberly A Lewis, Jose C Florez, Elena Flowers

**Affiliations:** Department of Physiological Nursing, University of California, San Francisco, CA 94143, USA; Bluestone Center for Clinical Research, New York University, New York, CA 10010, USA; Bluestone Center for Clinical Research, New York University, New York, CA 10010, USA; Department of Oral and Maxillofacial Surgery, New York University, New York, CA 10010, USA; Department of Physiological Nursing, University of California, San Francisco, CA 94143, USA; Department of Medicine, Center for Genomic Medicine and Diabetes Unit, Massachusetts General Hospital, Boston, MA 02114, USA; Programs in Metabolism and Medical & Population Genetics, Broad Institute, Cambridge, MA 02142, USA; Department of Medicine, Harvard Medical School, Boston, MA 02115, USA; Department of Physiological Nursing, University of California, San Francisco, CA 94143, USA; Institute for Human Genetics, University of California, San Francisco, CA 94143, USA

## Abstract

MicroRNAs (miRs) may contribute to disease etiology by influencing gene expression. Numerous databases are available for miR target prediction and validation, but their functionality is varied, and outputs are not standardized. The purpose of this review is to identify and describe databases for cataloging validated miR targets. Using Tools4miRs and PubMed, we identified databases with experimentally validated targets, human data, and a focus on miR–messenger RNA (mRNA) interactions. Data were extracted about the number of times each database was cited, the number of miRs, the target genes, the interactions per database, experimental methodology and key features of each database. The search yielded 10 databases, which in order of most cited to least were: miRTarBase, starBase/The Encyclopedia of RNA Interactomes, DIANA-TarBase, miRWalk, miRecords, miRGator, miRSystem, miRGate, miRSel and targetHub. Findings from this review suggest that the information presented within miR target validation databases can be enhanced by adding features such as flexibility in performing queries in multiple ways, downloadable data, ongoing updates and integrating tools for further miR–mRNA target interaction analysis. This review is designed to aid researchers, especially those new to miR bioinformatics tools, in database selection and to offer considerations for future development and upkeep of validation tools.

**Database URL** http://mirtarbase.cuhk.edu.cn/

## Introduction

The roles of micro-ribonucleic acids (miRs) in disease development are increasingly the subject of biobehavioral research (1). MiRs are small, single-stranded, non-coding ribonucleic acids (RNAs) between 17 and 25 nucleotides in length that operate at the post-transcriptional level to regulate gene expression (2). By binding to the 3ʹ untranslated region (UTR) of messenger RNAs (mRNAs), miRs can repress or degrade the mRNA targets and thus affect their translational efficiency (3). MiRs play key roles in many cellular processes, including development, metabolism, cell cycle, differentiation and death (2). Given their role in these biological processes, disruption of miR biogenesis or regulation can contribute to disease (4). MiR dysregulation has been investigated in different diseases, including cardiovascular disease, metabolic disorders, vascular diseases, neurological disorders and cancers (4, 5).

To define the regulatory role of miRs, it is essential to identify and validate interactions with mRNA targets (6). MiRs bind to the target mRNA through a region called the seed sequence, which is typically composed of six to eight nucleotides, and by doing so, regulate the translation of the target mRNA (7). This process is dynamic in that miRs can interact with hundreds of mRNA targets and mRNAs can be targeted by many miRs (8), rendering the study of miR–mRNA interactions challenging. Researchers have addressed this issue by using prediction methods and subsequent experimental validation of these miR–mRNA target interactions (MTIs) (9). As the number of studies of miRs has increased, a need for organizing the data about these interactions has arisen, and bioinformatic tools have become a useful way to manage and query these data. Generally speaking, bioinformatic tools can be grouped by the platform utilized, which is typically either a web-based service or a downloadable software and its related packages (such as R) (9). Web-based services tend to be user-friendly while downloadable packages allow for more flexibility in terms of data manipulation.

Target prediction algorithms can provide crucial information regarding MTIs. Some of these algorithms, however, are developed around the assumption of perfect pairing within the critical seed region. However, only a few nucleotides in the 3ʹ UTR of the target mRNA are required for sufficient base pairing to allow for miR regulation (10, 11). This property can yield different results between target prediction tools and lead to high false-positive predictions of MTIs (10, 11). Therefore, it is important to verify these interactions experimentally. There are direct and indirect methods of experimental validation of MTIs. Direct methods study the miR–mRNA pairs directly or introduce specific target sites that bind miR and reporter genes. These methods, which provide the strongest level of evidence for a functional MTI, include quantitative reverse transcription polymerase chain reaction (qRT-PCR), western blots and luciferase reporter assays (10). Indirect methods, which provide less robust evidence for a functional MTI, include high-throughput technologies to derive miR expression from altered mRNA or protein expression (10). These techniques include microarrays, ribonucleic acid sequencing, cross-linking and immunoprecipitation followed by sequencing and cross-linking ligation and sequencing of hybrids (4, 10).

Prior reviews either offered a broad overview of miR analysis tools, dedicating a small portion to target prediction and validation (4, 6, 12), or an evaluation of a few validated miR target databases (13). However, none of these prior reviews focused on experimentally validated databases with applications for *Homo sapiens*. The purpose of this paper is to identify, describe and compare currently available databases that curate experimentally validated data on human MTIs.

## Material and methods

**Figure 1.**
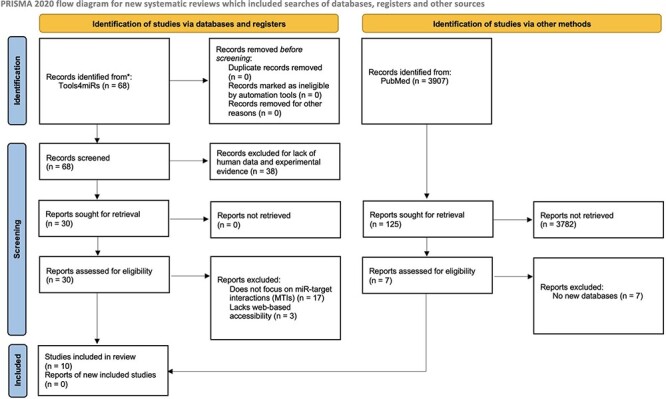
PRISMA flow diagram depicting the systematic selection process of databases for experimentally validated human MTIs.

The inclusion criteria for this review were web-based tool, experimentally validated target data, human miRs and a stated purpose of the tool was specifically cataloging miR–mRNA interactions. We used tools4miRs, an open-access platform with hundreds of tools dedicated broadly to miR analysis, to identify the total number of available databases (14). From there, we filtered for human miRs in the ‘organism-specific’ category and experimental evidence in the ‘data collection’ category. After reading through abstracts and descriptions of the databases, we selected miR databases that fit our inclusion criteria. One exclusion criterion was applied to tools that met the inclusion criteria but had an inaccessible database site. We also searched for additional miR target databases listed in PubMed using the search terms: ((((microrna*) OR (mirna)) OR (mir)) AND (target)) AND (database*). From the results, we sought reviews evaluating databases for human MTIs. We followed the Preferred Reporting Items for Systematic Reviews and Meta-Analyses (PRISMA) guidelines as depicted in Figure 1 (15).

The databases that met the inclusion criteria were evaluated for several attributes, including harboring only experimental evidence, including both experimental and computationally predicted targets, the total number of included miRs and target genes, the number of MTIs, web-based accessibility and the number of citations on Web of Science (WoS) until 2021. Assessment of these attributes was based on database websites and primary research papers on PubMed and/or WoS. When subsequent papers that described updates or advances in the tool were published, these papers were also reviewed for accurate information about database attributes. In order to verify the number of citations on WoS, the name of the database was first input as the search term; then the primary publication listing the database attributes and publications indicating updates to the database was checked for the total number of times they were cited. These citations were downloaded as an Excel file (Microsoft, Redmond, WA) and combined in R (version 4.1.1) for each database. Duplicates were removed based on the unique WoS identifier for each paper, resulting in the total number of citations.

For databases with downloadable data, the total number of miRNAs, target genes and MTIs was determined via R (version 4.1.1) using the packages tidyverse (version 1.3.1), janitor (version 2.1.0) and readxl (version 1.3.1). Each unique miRNA–gene pair was counted as a unique MTI to determine the total.

## Results

### Ten databases were identified for the validation of human MTIs

Figure 1 depicts the PRISMA flow diagram showing the process of selecting tools with experimentally validated human MTIs, whereby we identified 10 tools that met our inclusion criteria. All tools were available and regularly accessed between 1 September 2021 and 31 July 2022. Table 1 summarizes the relevant attributes of the identified experimental validation tools. These tools, in the order of the number of times cited, are miRTarBase (16–20), starBase/The Encyclopedia of RNA Interactomes (ENCORI) (11, 21), DIANA-TarBase (3, 22, 23–25), miRWalk (26–28), miRecords (29), miRGator (30–32), miRSystem (33), miRGate (34, 35), miRSel (36) and targetHub (37). The number of citations was derived from WoS records except in the case of miRSel, for which no results were found in WoS, and thus, we used records from PubMed. The top five databases provided an option to download data, which could then allow for the identification of exact numbers of human miRs, target genes and MTIs. One exception was miRWalk, whose downloadable information reflected MTI information from its prediction algorithm. Of note, there were discrepancies between the results obtained by searching the web interface and the results obtained from the downloaded datasets, and the reasons for this are not known. In addition to miRWalk, this was also the case for miRTarBase and DIANA-TarBase, whereby the number of MTIs derived from the downloaded data did not match those reflected on the database sites. Another inconsistency arose in miRTarBase between the updated version of the site (version 9.0) and the data that were available for download (version 8.0) within the aforementioned timeframe. From the seven databases with no downloadable option for the validated MTIs, only miRWalk displayed the total number of its validated entries in the database site. However, no information was given about the total number of miRs and target genes. This was also the case for miRGator, miRSystem, miRGate, miRSel and targetHub. The number of MTIs cataloged was the greatest in miRWalk, which derives its validated information from miRTarBase. miRWalk offers an enhanced feature building on the data resourced from miRTarBase by offering a predictive algorithm to ‘score’ the probability of an MTI interaction (a score of 1 is the highest probability) when specifying download parameters (28). Table 2 presents a summarized checklist of some useful features of the databases.

**Table 1.** Databases with experimental validation of human MTIs

Number	Database	Number of miRs	Number of target genes	Number of MTIs	Number of citations (WoS)	Experimental validation methods	Features	Database site
1.	MiRTarBase (10, 16–20)	2599	15 064	380 639	3217	CLIP-Seq, Luciferase assay, microarray, NGS, pSILAC, western blot	Offers different category options for browsing, e.g. by miR, by disease and by KEGG Pathway Downloadable data (from version 8.0) though database site is version 9.0 The Number of MTIs in version 9.0 as reflected on database site is 2 200 449.	https://mirtarbase.cuhk.edu.cn/∼miRTarBase/miRTarBase_2022/php/index.php
2.	starBase/ENCORI (11, 21, 38)	155	687	1286	2605	CLIP-Seq	Downloadable data Only uses high-throughput datasets	https://rna.sysu.edu.cn/encori/index.php
3.	DIANA-TarBase (3, 22, 23–25)	1084	20 820	422 662	1894	AGO-IP, biotin miRNA tagging, CLASH, CLEAR-CLIP, CLIP-Seq, ELISA, IMPACT-seq, microarrays, PAR-CLIP, proteomics, qPCR, Reporter genes, RIP-seq, RNA-seq, RPF-seq, TRAP, western blot, other	Offers various options for filtering results. Downloadable data Integrates other DIANA tools for miR analysis	https://dianalab.e-ce.uth.gr/html/diana/web/index.php?r=tarbasev8
4.	miRWalk (26–28)	2656	19 128	31 235 408	1737	CLIP-Seq, Luciferase assay, microarray, NGS, pSILAC, western blot	Updated twice a year Downloadable data for computationally predicted targets Experimentally validated entries from miRTarBase (# of MTIs reflected on miRWalk site)	http://mirwalk.umm.uni-heidelberg.de/
5.	miRecords (29)	303	1112	1748	986	ELISA, immunocytochemistry, northern blot, qRT-PCR, reporter assay, western blot, other	Downloadable data Not up to date; last updated in 2013	http://c1.accurascience.com/miRecords/download.php
6.	miRGator (30–32)	N/A	N/A	N/A	266	qRT-PCR, reporter assay, western blot	Experimentally validated data from miRecords, miRTarBase and TarBase Not available for download Not up to date	http://mirgator.kobic.re.kr/index.html
7.	miRSystem (33)	N/A	N/A	N/A	186	Reporter assay, western blot, qRT-PCR TarBase methods: CLIP-Seq	Integration of different databases Not available for download	http://mirsystem.cgm.ntu.edu.tw/
8.	miRGate (34, 35)	N/A	N/A	N/A	44	Not specified	Experimentally validated data from four databases: TarBase, miRTarBase, miRecords and OncomirDB Shows validation methodology, database name and PubMed ID Includes all isoforms of each gene Can download search results though no available download for all human miRs Query only by organism, gene and/or miRNA	http://mirgate.bioinfo.cnio.es/
9.	miRSel (36)	N/A	N/A	N/A	33 (based on PubMed results)	Not specified	Uses text-mining of biomedical literature (PubMed abstracts) to extract miR–target interactions Query via miR identifiers, gene and/or protein names, PubMed IDs, gene ontology (GO) terms Integrates information from TarBase, miRecords and miR2Disease Outdated links to TarBase	https://services.bio.ifi.lmu.de:1047/mirsel/
10.	targetHub (37)	N/A	N/A	N/A	10	Not specified	Experimentally validated information from miRTarBase	https://app1.bioinformatics.mdanderson.org/tarhub/_design/basic/index.html

The databases are listed in the order of the number of citations, with the most cited tools at the top.

AGO-IP, Argonaute immunoprecipitation; CLASH, cross-linking ligation and sequencing of hybrids; CLEAR-CLIP, covalent ligation of endogenous Argonaute-bound RNAs-cross-linking and immunoprecipitation; CLIP-seq, cross-linking and immunoprecipitation followed by sequencing; ELISA, enzyme-linked immunosorbent assay; IMPACT-seq, identification of miRNA recognition elements by pull-down and alignment of captive transcripts-sequencing; MTIs, miRNA–mRNA target interactions; NGS, next-generation sequencing; PAR-CLIP, photoactivatable ribonucleoside-enhanced cross-linking and immunoprecipitation; pSILAC, pulsed stable isotope labeling by amino acids in cell culture; qPCR, quantitative polymerase chain reaction; ; RIP-seq, RNA/ribonucleic acid immunoprecipitation sequencing; RNA-seq, ribonucleic acid sequencing; RPF-seq, ribosome profiling sequencing; TRAP, trapping by RNA *in vitro* affinity purification.

**Table 2.** Useful features of databases for validated human MTIs

Database	Downloadable data of all validated MTIs?	Constant updates/updated within last 5 years (2017–21)?	Includes MTIs validated via low-throughput methods?	Includes MTIs validated via high-throughput methods?	Allows queries through multiple methods (e.g. method, disease and KEGG Pathway)	MTI network visualization tool?
MiRTarBase (10, 16–20)	✔	✔	✔	✔	✔	✔
starBase/ENCORI (11, 21, 38)	✔	✔		✔		
DIANA-TarBase (3, 22, 23–25)	✔	✔	✔	✔	✔	
miRWalk (26–28)		✔	✔	✔	✔	
miRecords (29)	✔		✔	✔		
miRGator (30–32)			✔	✔		
miRSystem (33)			✔	✔		
miRGate (34, 35)			✔	✔		
miRSel (36)			✔	✔		
targetHub (37)			✔	✔		

#### Top databases feature frequent updates

The databases that indicate their most recent updates include miRTarBase, whose current version (version 9.0) was released in September 2021 (10), starBase/ENCORI with an update in November 2021 (38), TarBase, with its latest version released in 2017 (22), and miRWalk, which is updated twice a year (28). miRecords explicitly mentions that its last update was in 2013. Other databases such as miRGator, miRSystem, miRGate, miRSel and targetHub do not show evidence of constant updates, meaning some of these tools that derive MTI data from the top frequently updated databases have limited information on MTIs compared to the latter.

#### Databases catalog MTIs validated by low- and/or high-throughput techniques

Most databases report a combination of low- and high-throughput techniques applied to the validation of MTIs. A notable exception is starBase, which primarily uses high-throughput methods.

#### Supporting information is frequently provided

Among the highly cited and recently updated databases, starBase/ENCORI has the most descriptive help section with both graphical instructions and a glossary of terms (38). DIANA-TarBase also has a helpful graphical instruction page, but without additional text details (22). miRTarBase has limited user support/instructions but does include a graphical and text glossary of validation methods (10). miRWalk offers a ‘Frequently Asked Questions’ page to guide users on how to use the database including information on how to perform target searches, obtain information on only validated MTIs and interpret the ‘score’ of an MTI based on the algorithm used (28).

#### Overlap between database findings is variable

**Figure 2.**
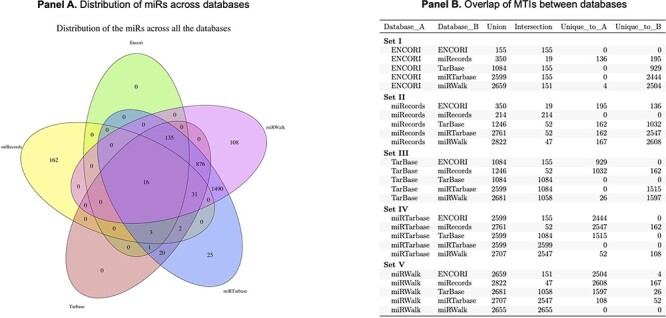
Panel A is a Venn diagram to show the total number of overlapping MTIs across the top five databases. Panel B is a table to show the number of total MTIs in each combination of two databases (Union), the number of MTIs overlapping between each combination of two databases (Intersection) and how many were unique to each database in combinations of two.

We performed an assessment of the overlap of miRs between all databases and found that only 16 miRs were identified in all of the top five databases (Figure 2). Several challenges were identified, including incorrect inclusion of non-human miRs and specificity of annotation of the −3p and −5p miR species. miRWalk and miRTarBase share the greatest overlap in web search-produced miRs (2707 in total), which is attributed to the fact that miRWalk derives validated information from miRTarBase. miRecords had the greatest number of unique miRs within the top five datasets; however, limitations include that the database is not frequently updated, and the site is not always accessible. No miRs were found to be unique to TarBase or ENCORI.

## Discussion

The purpose of this review was to evaluate the web-based tools available for the organization of experimentally validated mRNA targets of human miRNAs. With the increasingly recognized biological relevance of miRNAs to disease development, experimental validation of MTIs is necessary for the assessment of miRNA function. The utility of tools that curate validated evidence extends beyond the data cataloged because they can also be used to inform machine-learning methods for target prediction, especially those that use the validated data as a training set (23). Since the available databases are developed using different techniques and a range of purposes, it is necessary to comprehensively evaluate each tool’s attributes in order to determine which is most useful for a given application.

Ease of access in navigating the database is a key feature that is shared among some of the top databases. Though each database might define its user-friendly layout in different ways, maintaining flexibility in how users can perform queries is the most useful feature. The databases listed in Table 1 all allow users to search based on miRNA name and/or target gene name. Strength of MiRTarBase is that it features the option for searching by Kyoto Encyclopedia of Genes and Genomes (KEGG) Pathway, validation method, disease, PubMed ID and an advanced search option that allows one to input a list of miRs or target genes (20). Similarly, TarBase gives users multiple options to filter results by validation methods, regulation type (i.e. up, down or unknown), cell type and tissue type (22). A useful feature of the most cited databases gives the user the option to download the validated data, making it easy to manipulate further in programs such as R. The download feature was applied to this review, which allowed us to identify with certainty the total numbers of the miRNAs and genes included. Regarding citations, frequent use by the scientific community suggests that those tools are user-friendly and intuitive (39, 40).

We evaluated the number of miRs across the top five databases and found very little overlap. The lack of consistency between databases poses a major challenge for cross-validation between studies that utilized different databases to find MTI information. In some cases, the validated data from one database (e.g. MiRTarBase) is integrated into another database (e.g. miRWalk). While this might seem to provide validation, it is actually the replication of the same information across two tools. Overall, the lack of overlap between the top databases demonstrates a need for a more transparent and rigorous approach to cataloging validated human MTIs.

Among the tools included in this review, five were developed with a text-mining technique or manual curation to collect experimentally validated targets. These are miRTarBase, TarBase, miRecords, miRWalk and miRSel. However, miRWalk has since changed the way users can access validated targets by incorporating a filter that links to miRTarBase (28). Similarly, databases such as miRGator, miRSystem, miRGate and targetHub integrate data from one or a combination of miRTarBase, TarBase or miRecords. The latter were some of the earlier tools developed to catalog validated targets, TarBase in 2006 (22), miRTarBase in 2010 (19) and miRecords in 2008 (29) and therefore were foundational for subsequent tool development. While TarBase and miRTarBase continue to be updated regularly with the latest versions released within the last 5 years (2017–21), miRecords is no longer up to date with its last update in 2013. Other databases that undergo ongoing updates, also within the last 5 years, include starBase and miRWalk. This upkeep of databases is needed in order to catalog new MTIs as they are validated, making this an important consideration for researchers using these tools.

A key criterion in this review was that each tool has a stated purpose of cataloging MTIs with experimentally validated evidence. The lower throughput methods for experimental validation provide a stronger level of evidence for a functional MTI and are less likely to be falsely positive, whereas the high-throughput methods are a weaker level of evidence but generate a larger number of potential MTIs with a great likelihood for false positives but also excluding false negatives. Further inspection of the tools revealed that while all are designed to assess for experimental evidence of an MTI, others offer additional functionality. From our top five tools, miRTarBase and TarBase are explicit and intentional in their role of curating experimentally validated MTIs. However, starBase, the former version of ENCORI, was the second most cited tool according to WoS records, and besides describing MTIs, it also includes miRNA interactions with long non-coding RNAs, pseudogenes, circular RNAs and protein-RNA interactions (21). miRWalk and miRecords include both predicted and validated MTIs and in the case of miRWalk, it includes miRNA target site prediction using diverse algorithms (28). While an older version of miRWalk (27) hosted a ‘validated target module’ that allowed users to search by target gene, miRNA, BioCarta or KEGG Pathway, disease, cell line or proteins involved in miRNA processing, the option is no longer available and has been replaced by a miRTarBase filter for the validated targets as aforementioned (28). Within this review, we looked at the total number of times the database and/or primary database papers were cited, but this metric does not reveal how the tool was used. Thus, understanding the information that each tool harbors might be a better way for scientists to assess which database to use.

While some of the information that can be gleaned across databases is redundant (e.g. predicted and/or validated MTIs), some databases offer unique features. In order to attain the most comprehensive information about MTIs, some databases integrate other tools beyond target identification. Table 3 lists tools linked to each database, excluding those for target prediction. For example, MiRTarBase, with the highest number of integrated tools, includes 10 databases that provide additional information on miR regulation, disease associations and gene and miR expression profiles (20). An additional feature unique to miRTarBase is an option to visualize the regulatory network among miRs, regulators and gene targets. As part of DIANA tools, TarBase is interconnected with other tools such as DIANA-miRPath, for information on miR regulation in physiological molecular pathways, DIANA-miRGen for miR regulatory information and DIANA-plasmiR for information on circulating miR biomarkers (22). These integrated tools can make it easy for researchers to obtain a lot of information about an MTI from a single database and provide linkage to other tools for further miR analysis. Information obtained from one database with more limited applications can be entered into a more comprehensive database (e.g. MiRTarBase) to access linked tools and related information. In this way, the relative strengths and complementary functions of multiple databases can be leveraged in order to increase the overall utility of these tools for complex research questions.

**Table 3.** Tools integrated by the 10 databases for validated human MTIs

Target validation tool	Databases integrated by the validation tool
miRTarBase (10, 20)	CMEP, GEO, HMDD, miRBase, miRSponge, NCBI Entrez Gene, NCBI RefSeq, SomamiR, TCGA, TransMir
starBase (21)	Ensembl genome browser, GEO, miRBase, UCSC genome browser
DIANA-TarBase (25)	Ensembl genome browser, miRBase, miRGen, miRPath, plasmiR
miRWalk (28)	Disease Ontology, KEGG Pathway, miRBase, Reactome Pathways
miRecords (29)	NCBI Entrez Gene, NCBI RefSeq
miRGator (30)	GEO, Gene Ontology, KEGG Pathway, miRBase, SRA, TCGA
miRSystem (33)	BioCarta, Gene Ontology, KEGG Pathway, Interaction Database, Reactome
miRGate (34)	MiRBase, Ensembl
miRSel (36)	HUGO Gene Nomenclature Committee, miRGen, miRBase, NCBI Entrez Gene, Swiss-Prot Protein Database
targetHub (37)	MiRBase, NCBI Entrez Gene, UCSC Table Browser

Information about these tools was found via the most current database papers and database sites. Not included in this table are target prediction databases that are included in some of the target validation tools.

CMEP, Circulating MicroRNA Expression Profiling; GEO, Gene Expression Omnibus; HMDD, Human MicroRNA Disease Database; KEGG, Kyoto Encyclopedia of Genes and Genomes; NCBI, National Center for Biotechnology Information; SRA, Sequence Read Archive; TCGA, The Cancer Genome Atlas; UCSC, University of California, Santa Cruz.

There are challenges in this field, including infrastructure to sustain and support maintenance, updates and accessibility of tools. Funding to support the development of these tools may wax and wane or be limited in duration, which may impact perpetuity. A researcher may move to a new institution with a different web hosting service that can impact accessibility. Similarly, a researcher may retire with no succession plan for continuing to support a tool. In order to support the sustainability and continuous improvement of these tools, more centralized support may be needed (e.g. National Institutes of Health or other government-funded organizations’ initiatives or programs).

## Conclusion

The increasingly appreciated relevance of miRNAs in human diseases has led to a need for understanding their gene targets, and as a result, many databases have emerged to keep track of these interactions as they are discovered. Without a standardized approach to cataloging MTIs, databases differ in scope, organization and attributes; therefore, the aim of this review was to describe and compare them with the intention that it may provide researchers with a better understanding of the tool that best fits their needs. Databases with the option to download the data, which also happen to be the top cited validation tools, offer users flexibility in further manipulation of the data in programs such as R. Additionally, ongoing database updates are needed to ensure that the most comprehensive information on MTIs is discoverable. Features present in top cited databases like miRTarBase and TarBase that enhance the value of the database to users include providing various browsing options beyond searching by miR or gene target as well as integrated tools that further the analysis of MTIs. Tools can change over time in scope and purpose; thus, it is important to not only reference the primary papers and updates but also visit the database sites for a better understanding of what they can provide. As high-throughput sequencing data continues to accrue, the number of MTIs will increase apace, but low-throughput methods ensure strong evidence of direct miRNA–mRNA interactions. Scientists who seek to use these validation tools must then decide whether to use a tool like starBase, with only high-throughput methods, or miRTarBase and TarBase that are more frequently updated repositories with both high- and low-throughput methods. By providing a general overview of miR target validation tools with applications for *Homo sapiens*, this review hopes to aid researchers, especially those new to miR bioinformatic analysis, in choosing which database to use and inform the future development of tools with considerations offered within this paper.

Expanding the evaluation to more than web-based tools (e.g. R packages such as multiMiR) could reveal additional useful features for researchers interested in uncovering additional information on MTIs of interest. Future reviews could also create a comprehensive assessment of tool utilization. For tools that have both experimentally validated and predicted data, this could include making a distinction as to which datum was used in a given published study. There are a few databases (e.g. TarBase and miRTarBase) that have been consistently updated and offer new tools and features with each new release. In some cases, less commonly cited databases now link to these highly used and regularly updated tools. Looking forward, a small number of databases may emerge as the optimal tools across attributes that become the gold standard for MTI prediction and annotation.

## Data Availability

miRTarBase is available at http://mirtarbase.cuhk.edu.cn/ (10) starBase/The Encyclopedia of RNA Interactomes (ENCORI) is available at https://starbase.sysu.edu.cn/ or https://rna.sysu.edu.cn/encori/index.php (38) DIANA-TarBase is available at https://dianalab.e-ce.uth.gr/html/diana/web/index.php?r=tarbasev8%2Findex (22) miRWalk is available at http://mirwalk.umm.uni-heidelberg.de/ (28) miRecords is available at http://c1.accurascience.com/miRecords/download.php (29) miRGator is available at http://mirgator.kobic.re.kr/index.html (31) miRSystem is available at http://mirsystem.cgm.ntu.edu.tw/ (33) miRGate is available at http://mirgate.bioinfo.cnio.es/ (34) miRSel is available at https://services.bio.ifi.lmu.de:1047/mirsel/ (36) targetHubis available at https://app1.bioinformatics.mdanderson.org/tarhub/_design/basic/index.html (37)
